# Isoform- and Paralog-Switching in IR-Signaling: When Diabetes Opens the Gates to Cancer

**DOI:** 10.3390/biom10121617

**Published:** 2020-11-30

**Authors:** Pierluigi Scalia, Antonio Giordano, Caroline Martini, Stephen J. Williams

**Affiliations:** 1 Sbarro Institute for Cancer Research and Molecular Medicine, Temple University, Philadelphia, PA 19122, USA; giordano@temple.edu (A.G.); tuf88004@temple.edu (C.M.); sjwilliamspa@comcast.net (S.J.W.); 2 ISOPROG-Somatolink EPFP Network, Functional Research Unit, Philadelphia, PA 19104, USA and 93100 Caltanissetta, Italy; 3 Department of Medical Biotechnologies, University of Siena, 52100 Siena, Italy

**Keywords:** *IR*: insulin receptor, *IGF*: insulin-like growth factor, *HIF:* hypoxia-inducible factor, *Isoform:* for the scope of this review, the term isoform is restricted to products of alternatively spliced coding genes, *Paralog*: the product of gene variants with high sequence similarity encoded by duplicated genes in the genome, MAPK-ERK: Mitogen-activated protein Kinase-Extracellular-signal-regulated Kinase

## Abstract

Insulin receptor (IR) and IR-related signaling defects have been shown to trigger insulin-resistance in insulin-dependent cells and ultimately to give rise to type 2 diabetes in mammalian organisms. IR expression is ubiquitous in mammalian tissues, and its over-expression is also a common finding in cancerous cells. This latter finding has been shown to associate with both a relative and absolute increase in IR isoform-A (IR-A) expression, missing 12 aa in its EC subunit corresponding to exon 11. Since IR-A is a high-affinity transducer of Insulin-like Growth Factor-II (IGF-II) signals, a growth factor is often secreted by cancer cells; such event offers a direct molecular link between IR-A/IR-B increased ratio in insulin resistance states (obesity and type 2 diabetes) and the malignant advantage provided by IGF-II to solid tumors. Nonetheless, recent findings on the biological role of isoforms for cellular signaling components suggest that the preferential expression of IR isoform-A may be part of a wider contextual isoform-expression switch in downstream regulatory factors, potentially enhancing IR-dependent oncogenic effects. The present review focuses on the role of isoform- and paralog-dependent variability in the IR and downstream cellular components playing a potential role in the modulation of the IR-A signaling related to the changes induced by insulin-resistance-linked conditions as well as to their relationship with the benign versus malignant transition in underlying solid tumors.

## 1. Introduction

Insulin resistance has been associated with a variety of insulin receptor (IR) signaling defects [1,2]. A number of such defects are affected by the expression of specific IR isoforms [3]. In fact, two isoforms of the IR exist—IR-A (ex 11−) and IR-B (ex 11+)—as a result of the alternative splicing of exon 11 [4], conferring to the first high affinity to IGF-II [5]. Most recently, the IR receptor has gained attention as a receptor, mediating specific cancer- and diabetes-related responses, which are instead not controlled by the IGF-I receptor [6,7,8]. Hyperinsulinemia is a compensatory condition induced by insulin resistance in obesity and type 2 diabetes. Under these conditions, the chronically increased insulin levels associate with increased cancer risk [9,10] and to parallel increase levels in IR and Insulin-like Growth factor I (IGF1R) [11]. The IR isoform expression status in these cases is a potentially relevant type of information due to the fact that the increased IR-A/IR-B ratio correlates to higher proliferation and lower metabolic response [12,13]. The finding that cancer-secreted IGF-II can activate different targets compared to those activated by insulin through activation of the insulin receptor isoform A [14,15] opens-up a novel scenario for the role of this ligand/receptor axis. This is particularly relevant in those conditions where they are co-expressed, such as during the pathogenic process linked to insulin-resistant states and cancer. In this review, we focus on the role of the IR-A and its activation by IGF-II in the context of insulin-resistance-associated states, namely obesity and diabetes. This review also provides a scenario where isoform and/or paralog variants of its upstream and downstream network are potentially involved in the benign versus malignant transition occurring in solid cancers with underlying insulin-resistant states.

## 2. The IR Isoform A: A Molecular Discriminant Linking Obesity and Diabetes Type 2 to Cancer?

**Figure 1:**
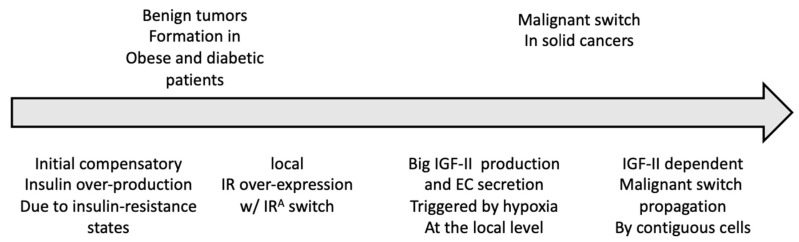
Proposed model for the role of insulin receptor (IR) and insulin growth factor (IGF)-II splicing variants in solid tumor progression from insulin-resistant/hyperinsulinemic states to overt malignancy.

Among sequence defects linked to diseases, almost half of these defects can affect splicing [16]. In the case of the IR gene, the alternative splicing of exon 11 has been found to be affected by development, aging, and cell type conveyed by specific ligand-generated signals. For example, in pancreatic beta cells, insulin has been found to favor exon 11 retention through RAS-MAPK/ERK pathway-induced upregulation of a specific exon-retention splicing factor (SRSF1) [17]. Nonetheless, since the first description of IR isoforms [4], due to their similar ability to bind insulin and mediate well-described insulin metabolic actions [18], no biological role was assigned to these two IR variants for almost a decade. Indeed, the first findings, supporting a different biological role of the two IR isoforms, occurred in the late nineties [5] when human IR with missing exon 11 (IR-A) had been found to mediate the effects of IGF-II, formerly thought to be just an alternative ligand and activator of the IGF1R (along with IGF1). The relevance of that study was not only to have disclosed a biological difference for the two IR isoform variants (which are equally activated by insulin) but to have provided a clear cancer-promoting role to the IR, previously thought to be linked to cancer only due to metabolic requirements. Interestingly, in spite of a general reduction in IR levels in insulin-resistant states [19], the IR-A/IR-B ratio is increased in several tissues [20], supporting the scenario proposed in Figure 1 for the role of the IGF-II-IR-A axis in linking obesity/type 2 diabetes and cancer development. Indeed, a number of signaling proteins involved in IR signaling undergo either paralog and or isoform switch in cancer [21,22,23]. Although, at present, our knowledge on the role of alternative splicing and paralog genes switch of signaling components involving insulin-resistance-associated conditions is limited (since most studies focus on full-length native proteins) (Table 1), the growing evidence linking hypoxia to differential isoform and/or paralog expression may provide the rationale to functionally connect these gene product variants with specific abnormalities observed in obese and type 2 diabetic patients in light of the chronic exposure of tissues to hypoxia (e.g., in adipose tissue) occurring under these conditions [24,25]. Additionally, a further connection between insulin-resistant conditions and cancer is the link between hypoxia and new vascularization in tridimensionally growing tumor formations [26,27], which constitutes a key requirement for tumors’ malignant transition. Possible mechanisms, functionally linking hypoxia in insulin-resistant conditions to cancers, are further discussed in the chapter below. Current knowledge of IR and IR/IGF1R heterotetrameric hybrids in a wide number of normal and pathological tissues provide a scenario spanning from the expression of IR isoforms (IR-A and IR-B) and IGF1R homo-dimers to the formation (by random disulfide bonds formation) of hybrid IR-IGF1R hetero-tetrameric receptors (reviewed in [13]). The prevalence of IR-A monomer among IR dimers and of IGF1R monomer among IR/IGF1R hetero-dimers (HR) can cause downstream signaling diversification compared to the homodimeric IR and IGF1R forms [28]. Hybrid receptors (HR), in the full spectrum of possible cellular combinations (including IR-A/IR-B, IR-A/IGF1R, and IR-B/IGF1R), account for half of the overall IR and IGF1R cumulative normal tissues content. HRs also co-exist in a variety of pathologic conditions [13]. For example, in diabetes, HRs are found to increase in muscle and fat tissues, and this increase correlates with reduced insulin sensitivity in vivo [3,13]. According to some authors, this could be possibly due to the fact these receptors preferentially bind to IGF-1 than insulin [3,13]. Our current hypothesis support that under the condition of increased IR expression, any increase in the IR-A/IR-B ratio and the induction of local production in IGF-II in response to chronic and/or local hypoxic stimuli can provide the micro-environment advantage towards the onset of overt cancerous foci (see Figure 1). The growing findings, showing differential IGF-II targets with cancer-promoting function activated through the IR-A but not via insulin on the same receptor [15,29], nor by the IGF1R [30], further support such view and point at the IR-A expression as a possible functional discriminant between diabetes and cancer and a valid biomarker for cancer along with other established biomarkers.

## 3. The IGF-II/IR^A^ Signal: A Booster for Malignancy in Diabetic and Pre-Diabetic Conditions?

The expression, secretion, and autocrine stimuli of IGF-II are common and well-established features in both the epithelial (carcinoma) and mesenchymal (sarcoma) types of solid cancers [30,52,53,54,55]. The overexpression of IGF-II in mammals is partially due to p53 deregulation [56,57,58]. Although IGF1 has been proposed as a tumor-promoting factor due to its established oncogenic effects exerted through its receptor (IGF1R) in vitro and in engineered mouse models [59,60], a substantial number of published evidences [61] point at IGF-II as the insulin-like growth factor used by most cancers and produced by malignant cancer cells in vivo. This view is further supported by the fact that many previous studies trying to correlate systemic bloodstream levels of IGF1 and cancer have remained inconclusive [62,63,64], while, on the contrary, high IGF-II blood levels have been often reported to be associated with a variety of solid cancers [63,65,66]. This is thought to be caused by secretion by malignant cells in the local microenvironment with possible spill-over in the bloodstream at detectable or overtly high levels depending on the size of the tumor, directly correlated to the number of IGF-II-producing cells. For these cancers, it has been proposed the name of “IGF-2-omas” to highlight this commonly shared feature [61]. It is important to note that when expressed in cancer, IGF-II is secreted as partially-processed forms generated by retention of the E domain, which bears O-glycosylation sites, allowing enzymatic attachment of glycosyl groups, conferring in this way its characteristic high molecular weight and the name of “big-IGF-II” [31,32]. These forms have been shown to retain full ability to stimulate their tyrosine kinase receptors, while they seem refractory to Insulin-like Growth Factor Binding Proteins (IGFBPs) binding [32,33]. This is an important finding since it provides a mechanism for the potential increase of its bioavailability in the tumor microenvironment by escaping the control of IGFBPs on the mature or physiologically processed ligand. The specific involvement of IGF-II in malignant tumor transition has been established in the Rip1Tag2 mouse model developed by Douglas Hanahan in the mid-eighties [67,68,69]. In this engineered mouse model, pancreatic carcinoma develops on hyperplastic formations driven by a Simian Virus 40 (SV40)-large T antigen transgene, targeting insulin-producing pancreatic cells upon sequential spontaneous activation of the IGF-II gene, which correlates to the tumor vascular switch [52]. The observed IGF-II angiogenic switch in this mouse model has been linked to activation of the IR-A but not to the IGF1R [70]. Another study showed that the IR-A was highly expressed in tumor vasculature and affected both endothelium growth in vitro and tumor angiogenesis in vivo [71]. The IGF-II/IR-A axis as a required mechanism towards malignant progression has been further strengthened by both the failure of IGF1R drug inhibitors in clinical trials [72] as well as by the demonstration of IR-A expression as a critical factor for the intrinsic resistance to the proposed therapeutic block of the IGF1R [6,73,74,75,76].

## 4. Hypoxia and Paralog and Isoform Switch in the Insulin-Resistant-Linked States and Cancer

**Figure 2:**
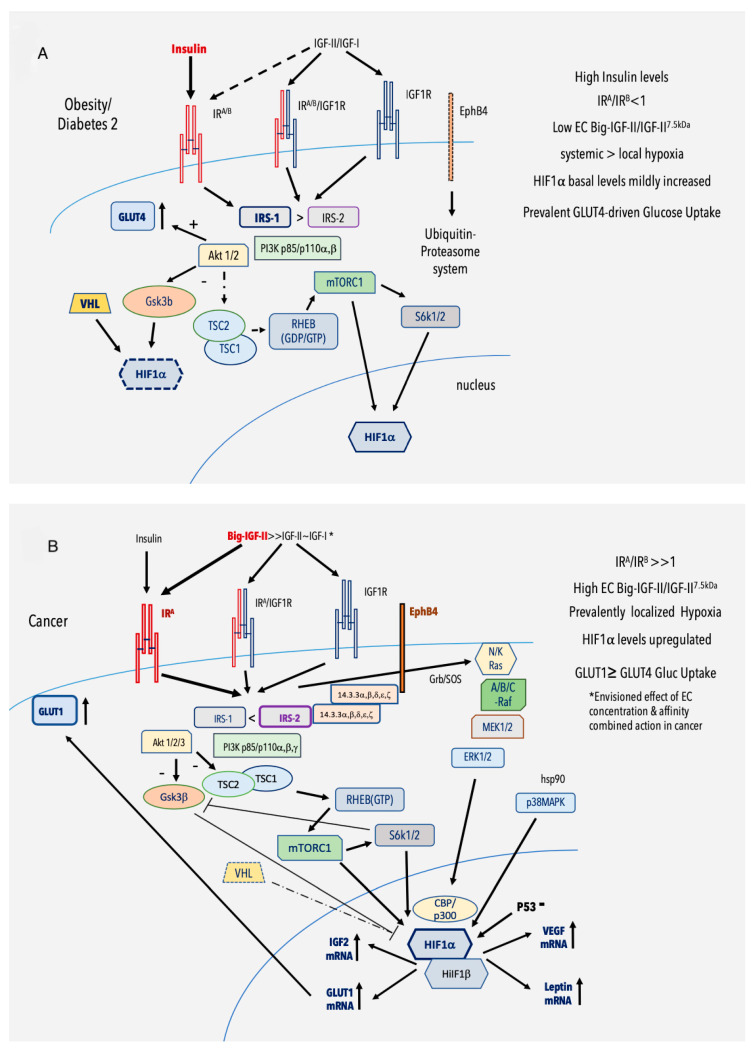
Working model for the regulation of Hypoxia-Inducible Factor (HIF)-1 by the IGF/IR system under insulin-resistant-associated conditions (**A**) and in cancer (**B**). (**A**) Effects of generalized and chronic hypoxic stimuli on HIF1α cellular content by the IR/IGF signals (such as under insulin-resistant-associated conditions). (**B**) Effects of marked and localized hypoxic stimuli on HIF1α by the IGF/IR system and signaling (such as in malignancy). Explanation in the text.

Tissue hypoxia has been associated with obese and diabetic patients [77]. In early solid cancer stages, hypoxia triggers angiogenic signals in developing solid tumors [69,78,79]. Paralogs, typically generated by gene duplication during genome evolution [80], can be differentially affected by hypoxia [81], including those directly involved in cell proliferation and metabolism [34]. Interestingly, paralog dependency and alternative splicing are functionally interconnected in cancer [82]. Hypoxia, per se, is a well-recognized trigger for alternative splicing for a variety of cellular factors involved in a number of cancer-promoting functions [83,84]. One of the first demonstrations of the role of low extracellular pH (a key effect of hypoxia) on exon skipping had been shown for Tenascin-C, an extracellular matrix protein overexpressed in cancer [85]. This effect has been widely studied for the IR where the retention of exon 11, leading to the expression of its full-length (or IR-B) isoform, is linked to the abundance in the IR pre-mRNA spliceosome of SRSF1 and the parallel low abundance of hrRNP-A1 [86] and, independently, to the exon-inclusive effect of SRp20, SF2/ASF [87], Mbnl1 [88], and Stau1 [89,90]. On the contrary, IR exon 11 skipping seems to be favored by the relative abundance of CUG-BP1 [87] and SRSF3. Interestingly, in mice, liver-specific deletion of SRSF3 has triggered hepatocarcinoma by inducing both IR-A and igf2 expression [91], highlighting the role of the IGF-II/IR-A axis in the liver tumor. Indeed, the link between hypoxia through HIF-1α, IGF-II, and VEGF expression has been shown to bear a reciprocal relationship [6]. In fact, in a hypoxic environment, HIF(1 and 2) can induce both IGF-II and VEGF expression [51,92], while other studies have also reported the ability of IGF-II to induce both HIF-1 and VEGF expression [93,94,95]. Under the same conditions, p53 deregulation has been shown to upregulate HIF-1α by protein stabilization and increase VEGF [96] in line with the above-cited effect on IGF-II. Indeed, p53 deregulation via intron retention nonsense-mediated decay (NMD) [83] can justify per se direct IGF-II upregulation due to the release of its inhibitory effect on IGF-II transcription [56,57,58]. These mechanisms strengthen the above link between hypoxia, IGF-II, and early-onset blood-vessel formation in cancer, along with a potential synergistic action with other angiogenic factors and adipokines released under the same circumstances. In regards to the components targeted downstream to the IR/IGFR receptor system, which can be affected by hypoxia, IRS2 (but not IRS1) is upregulated in breast cancer cells, where it enhances their survival and invasion activities [97]. The PI3K/AKT pathway upregulates HIF-1α, confirming positive feedback between HIF-1α targets and the IR-IRS1/2-PI3K-AKT axis. Specifically, published evidences suggest that HIF-1α phosphorylation by GSK3β triggers its Fbw7 and SCF complex-mediated degradation [98]. Interestingly, prolonged hypoxic stimuli in HepG2 has caused AKT deactivation and GSK3β activation, resulting in increased phosphorylation, and the degradation of HIF1α [99] indicates that this negative feedback may play a role in vivo and could be partially desensitized or deranged in chronic hypoxic conditions, such as those discussed herein. Noteworthy, a specific role for GSK3 paralogs in conditions, such as obesity and diabetes, has not yet emerged. The PI3K/AKT pathway regulates HIF-1α post-transcriptionally via mTORC1 [100,101]. This can occur under hypoxic conditions via S6K1-mediated phosphorylation and by acting directly on HIF-1 protein synthesis through 4E-BP1 [44]. Under the same conditions, mTORC1 can induce HIF1α transcriptional activation via STAT3 [102]. mTOR is also regulated by hypoxia via hypoxia-inducible REDD1. Such effect requires a functional TCS1/TCS2 complex [103]. It is worth noticing that HIF1α isoform 3 does not express the 92 C-terminal amino-acids bearing one of its two trans-activation domains, localized, respectively, at the N- and C-terminal level. This has practical implications since this region contains a post-transcriptionally-modified (PTM) residue targeted by the p38 MAPK pathway, which has been involved in HIF-1α nuclear accumulation under stress responses involving hsp90 [98]. Since these key pathways are activated by the IR signal, the balance between molecular discriminants in terms of circumstantial co-expression (by both protein stability and gene transcription) and isoform expression (affecting sensitivity to the same signals) can play a consistent part in the qualitative response exerted by hypoxia under hyper-insulinemic states (obesity and type 2 diabetes) as well as by IGF-I and II paracrine-stimuli and IGF-II autocrine loop during cancer progression and malignant transformation. As it relates to metabolic processes, at least 14 family members of glucose transporters (GLUT) have been described and/or characterized so far [104]. The understanding of glucose transporters (GLUT) biology might indeed offer a potential parameter for evaluating the degree of a metabolic shift occurring between benign growth in insulin-resistant states and overt malignant transformation. The relative expression of GLUT1 and GLUT4 in tissues, independently from the potential contribution of other paralog family members, holds functional consequences on glucose uptake and metabolism in cancer [19]. This is consequential to the fact that while GLUT4 is mainly expressed in muscle and fat tissues and exquisitely regulated by insulin [105], GLUT1 is ubiquitously expressed in cells and tissues [106] and is not regulated by insulin but induced by hypoxia at the transcriptional level [107,108] and is overexpressed in cancer [109,110,111,112]. Therefore, preferential and/or alternative expression of hypoxia-induced insulin-independent GLUT1 versus insulin-dependent GLUT4 can lead cells and tissues metabolism to hormonal independence. Interestingly, IGF-II is upregulated by hypoxia [51,95]. Since IR-A is activated by IGF-II, this offers an alternative mechanism for GLUT4-dependent glucose uptake under hypoxic states, such as in diabetes and cancer. Ultimately, the finding that six putative alternatively spliced forms of GLUT1 and four isoforms of GLUT4 (two of which confirmed) have been described (see Uniprot database) raises logical questions towards understanding their specific role and regulation under the scenarios discussed herein. These findings have been used in order to generate a working scenario summarized in Figure 2A,B, focusing on HIF-1α regulation by the IGF/IR system and signaling in the context of insulin-resistant states (2A) and cancer (2B). The finding that obesity determines a chronic hypoxic state in adipose tissue and several other tissues, which is common to insulin-resistant conditions, has been widely reviewed [24,77,113,114]. Under such circumstances, fat tissue and macrophages locally release a variety of known factors termed, under this context, pro-inflammatory adipokines. These so far have included leptin, adiponectin, PAI-1, VEGF, TNFβ, IL-1, IL-4, IL-6, IL-8, MCP-1, MIF, CCL-2, CCL-5, TGFα, IFNγ, having in common the fact of being induced by HIF1α under hypoxic states [115] and to be able to induce angiogenesis (reviewed by Ye) [93]. Ultimately, the overall effect of hypoxia found in obesity and insulin-resistant states on alternative splicing of critical signaling molecules with intrinsic cancer-promoting features is emerging as a wide and still unexploited framework, functionally linking such conditions with cancer development in vivo (see Table 1). It also offers a specific scenario to understand the specific effects of the IGF-II/IR-A axis and the parallel cytokine and receptor tyrosine kinase network towards solid cancers’ malignant transition.

## 5. Isoform- and Paralog-Switching in Benign to Malignant Phenotype Progression

Isoform switching based on alternative splicing has been commonly observed in cancer [21,22,23]. However, the specific role of such a mechanism on the overall progression towards overt cancer is only starting to emerge. For example, the PI3K catalytic subunit (p110) is expressed in four variants (alpha, beta, delta, and gamma), each bearing specific features in terms of cellular transformation ability [36,116]. PI3K110-alpha is a selective mediator of growth factors-induced endothelial cell proliferation [37]. AKT paralogs have been found to modulate the IR signaling in that AKT1 and 2 are key to glucose metabolism [117], while AKT3 is involved in postnatal brain development [118]. Furthermore, AKT1 and 2 seem to bear distinct or diversified effects in cancer progression [38]. The overall findings emerging in terms of extracellular activation and diversified signaling in metabolism and cancer for AKT (reviewed in Gonzales and McGraw [39]) will play a key role in further defining their exact involvement in modulating the IR signal during the malignant transition occurring in obese and overtly diabetic patients. Likewise, it will be relevant to identify the role of paralogs and/or alternatively spliced mTOR intrinsic regulatory components in its two major cellular complexes—mTORC1 (Raptor-driven) and mTORC2 (Rictor-driven) [44]—towards affecting the IR/IGF1R signaling in obesity/diabetes and cancer. For example, mSIN1 is an exclusive mTORC2 binder, which has three different isoforms. Interestingly, only two of the three isoforms complexed with mTORC2 are sensitive to insulin stimulation [119]. S6K1 is a known phosphorylative target activated by mTOR [120]. S6K1 displays three isoforms produced by alternative splicing, respectively, generating a p85, p70, and p31 proteins. Interestingly, p70 S6K1 but not p85 or p31 S6K1 are regulated by TSC2/mTOR towards nuclear translocation [42]. This is relevant since, as we mentioned earlier, HIF-1α is a target of mTORC1 through S6K1 under hypoxic conditions [102]. In this context, S6K1 has been shown to directly regulate GSK3 under mTOR-dependent feedback inhibition of AKT [121], which is a known GSK3 phosphorylative inhibitor [122]. This feedback signal has potential consequences on the pathological scenarios discussed herein due to the direct effect of GSK3 on HIF-1 degradation (reviewed in [98]). GSK3 paralogs are similarly regulated by growth factors-initiated signals via inhibitory phosphorylation at Ser-21 (GSK3α) and Ser-9 (GSK3β), respectively [40,123]. Although in vivo studies in knock-out (KO) animals have shown tissue-specific effects [124], and, in addition, GSK3α has shown to have a paralog-specific role in Fragile-X pathogenesis [41], their difference in cancer development is still a matter of investigation. Nonetheless, the deregulation of GSK3 paralogs’ anti-tumor suppressing activity driven by AKT hyperactivation [125] might play a role in linking benign versus overt malignant behavior at the cellular level. 14-3-3 proteins constitute a group of phospho-binding proteins made of seven isoforms, forming homo and heterodimers differentially localized in all cellular compartments and promoting a number of regulatory and modulatory actions [126,127,128,129,130,131]. 14-3-3 proteins bind hundreds of serine/threonine phosphorylated cellular proteins as part of specific phospho-signaling cascades, of which they determine the subcellular localization (reviewed in [132,133]. They have been found to bind both the IGF1R and the IR as well as to IRSs and to modulate GLUT4-mediated glucose transport upon insulin stimulation (reviewed in Chen et al. [134]). 14-3-3 proteins display both isoform-specific functions as well as partially redundant functions in their interaction with specific cellular targets [45,135,136]. Among the 14-3-3 targets of the IR signal involved in the physiologic cytoskeletal remodeling and cellular motility as well as part of the invasive oncogenic behavior, the small GTPases Rho and Rac1 have been found to interact with multiple 14-3-3 paralogs [137]. 14-3-3 isoforms dimers play a critical role in the organization and modulation of B-Raf dimers as well as the B-Raf/MEK complex [138]. B-Raf is a critical component of the RAS-MAPK pathway activated by most membrane receptor tyrosine kinases (RTKs), including the IR [139,140]. B-Raf alternative splicing is known to play a role in overcoming the chronic block to V600E B-Raf mutant in melanoma and other solid cancers (reviewed in Scalia et al. [48]). Interestingly, under such circumstances, a double block of MEK and IGF1R seems to overcome some isoform switch-dependent blocks [141]. Such a strategy was applicable only to a subgroup of the patients with B-Raf block resistance since the IGF1R expression was found just in one-fifth of them as part of a MAPK reactivation rescue pathway [49]. Since IGF1R and IR-A have been found co-expressed in solid cancers, and IR-A has been found over-expressed in a number of cancers with low IGF1R expression [26,33], it is feasible that in those cases where there is a mutated B-Raf isoform switch, this would associate with MAPK reactivation and low IGF1R content. In this scenario, the block of the IGF-II/IR-A signal could prove effective towards blocking and/or reverting the fast progression linked to B-Raf mutant isoform switch in solid cancers. HIF1 cellular content is regulated by hypoxia via the Von Hippel Landau (VHL) tumor suppressor protein, which, under decreased oxygen conditions, stabilizes the protein by preventing its ubiquitin-mediated degradation [141]. Among HIF-1α targets relevant to malignant cancer transition, we have already pointed, herein and elsewhere [6], at its ability to upregulate VEGF and IGF-II. Interestingly, HIF-1 and its α/β heterodimer have also been found to transactivate the leptin gene [142], which has shown to bear cancer-promoting effects [143]. A peculiar example of the role of isoform switch by the insulin receptor signaling with direct possible implications on cancer malignant transition regards the regulation of EphB4 protein levels in cancer cells by the autocrine IGF-II/IR-A signaling axis. EphB4 is a member of the Eph/Ephrin tyrosine receptor kinase family involved in the formation of blood vessels during embryonal development [144]. It is typically expressed in venous endothelial cells and interacts at the extracellular level with its binding partner EphrinB2, which is expressed in arterial endothelial cells in order to establish a forward and reverse signal, conveying its pro-angiogenic and differentiative stimuli [145,146]. EphB4 is ectopically expressed in a variety of solid cancers where it participates in a number of oncogenic effects, including angiogenesis, invasion, and metastasis [147,148,149]. We recently demonstrated that EphB4 bears a phospho-inhibited degron in its last C-terminal amino-acid residues [30]. Specifically, we identified EphB4 tyrosine 987 as the target of the IGF-II/IR-A (but not IGF-II/IGFR) signal and found that phosphorylation at this site associates with stabilization of the EphB4 protein along with lower ubiquitination levels, while IGF-II/IR-A signal neutralization leads to tyrosine 987 dephosphorylation and overall rapid protein degradation. Such mechanism is one of the first described circuits, associating directly the IGF-II/IR-A signal to the tight control of a protein, which bears angiogenic, invasive, and metastatic actions, and not to general cellular growth and mitogenic actions, therefore potentially constituting a specific malignant transition checkpoint. According to this model, the local autocrine IGF-II/IR-A signal would provide a second necessary hit towards overt malignant phenotype transition. This model is in agreement with the one postulated by studies in Rip1Tag2 mice where this axis has been shown to be mandatory for the transition of benign pancreatic tumors to highly vascular and invasive carcinoma [52,70].

## 6. Conclusions and Perspectives

Overall, the biological impact of paralog and isoform switching in IR in upstream regulatory (IGF-II) and downstream signal modulators under those clinical conditions sharing insulin-resistance, hyperinsulinemia, and chronic hypoxic states (obesity and type 2 diabetes) along with their link to cancer development is just emerging. A clear scenario suggested by evidences reviewed herein implies that the IR isoform A specific expression constitutes an early driver for the cancer-malignant transition. Although the IR and its hybrid receptor with the IGF1R can be activated by both insulin and the IGF (I and II) ligands, activation of its short isoform (IR-A) by IGF-II provides a selective malignant transition signal, functionally and sequentially linking cellular and tissue overgrowth commonly seen in hyper-insulinemic states, such as in obesity and type 2 diabetes. Here, the role of the autocrine signal exerted by big IGF-II is emphasized, secreted by cancerous and pre-cancerous cells in response to p53 deregulation and/or in response to local hypoxia. The overall result of paralog- and isoform-specific signaling is overt cancer growth characterized by malignant features, such as intra-tumoral blood vessel formation along with local invasion and metastasis. The isoform-specific and/or paralog expression-dependent intracellular signals discussed herein may be affected by the same inducers (e.g., splicing factors), leading to the IR-A and big IGF-II isoforms production under the same conditions (marked local hypoxic stimuli in tissues). Therefore, targeted transcriptomic profiling of single cells and homogeneous tissue components from tissue biopsies aimed to detect isoform switch events linked to IR-A-dependent signaling in patients with familiarity for solid cancers and insulin-resistant-associated conditions (namely obesity and type 2 diabetes) could soon offer a complementary strategy for prediction, prevention, and early personalized interventions in such patients.

## Figures and Tables

**Table 1:** Alternatively spliced and gene paralogs involved in IR signaling.

Prot/Complex Name	Underlying Process	Variant	UniProt	Isoform Biologic Advantage	References
Insulin receptor	Exon 11 skipping	IR-A	P06213-2	Confers high binding affinity to IGF-II	[5,14]
IGF-II	Alternative splicing (Exon retention)	1-104 1-128 (cleaved)	P01344	Confers O-glycosylation site; provides variants escaping IGFBP-regulation	[31,32] [32,33]
RAS	Gene duplication	N-RAS H-Ras K-RAS	P01111 P01112 P01116	Differential signaling	[34,35]
PI3K-p110	Alternative splicing	P110 a, b, d, g	α: P42336 β: P42338 Δ: O00329 γ: P48736	Confers RAS and ERK dependency (b,g) and angiogenic properties (a)	[36] [37]
AKT	Gene duplication	Akt1 Akt2 Akt3 paralogs	AKT1:P31749 AKT2:P31751 AKT3:Q9Y243	Metabolic and cellular proliferation (Akt1&2). Brain development (Akt3)	[38] [39]
GSK3	Gene duplication	GSK3a GSK3b paralogs	GSK3-α:P49840 GSK3-β:P49841	Master regulator with target suppressor function in a variety of conditions. GSK3-a involved in Fragile-X pathogenesis in mice model	[40,41]
S6K1	Alternative splicing	P70, p85 and p33 isoforms	P70: P23443 P85: P23443-1 P33: P23443-2	Involved in protein translation and mRNA maturation	[42,43]
mTOR	Differential mTORC ½ assembly of co-expressed paralogs	mTORC1 (mTOR+Raptor +mLST8+Deptor +PRAS40) mTORC2 (mTOR+Rictor +mLST8+Deptor +mSIN1+Protor1/2)	mTOR: P42345 Raptor: Q8N122 mLST8: Q9BVC4 Deptor: Q8TB45 PRAS40:Q96B36 Rictor: Q6R327 Deptor: Q8TB45 mSIN1: Q9BPZ7 Protor1:P85299	Differential assembly confers mTOR conditional sensitivity to Rapamicin (mTORC1 sensitive, mTORC2 insensitive)	[44]
14-3-3	Gene duplication	7 paralogs	β: P31946 γ: P61981 ε: P62258 ϴ: P27348 ζ: P27348 σ: P31947 η:Q04917	Intracellular phospho-target diversification	[45]
RAF	Gene duplication	A-RAF, B-RAF, C-RAF paralogs	A-RAF:P10398 B-RAF:P15056 C-RAF:P04049	Partially redundant and forming dimers	[46,47]
B-RAF	Induction by B-Raf chronic pharmacologic block	FL and P61 (WT and V600E) isoforms	P15056	P61 confers resistance to Vemurafenib block	[48,49,50]
HIF-1α	Induction by hypoxia, prolonged serum starvation and IGF-II	3 isoforms	Q16665 Q16665-2 Q16665-3: A8MYV6	Triggers IGF-II production during local tissues. Hypoxic and GF-deprivation conditions	[51]

Acronyms. RAS: Rat-Sarcoma gene product; PI3K: phosphatidyl-Inositol 3 kinase; AKT: AKR-Thymoma-8 retrovirus-derived serine-threonine kinase; GSK3: Glycogen Synthase Kinase 3; S6K1: (ribosomal protein) “S6” Kinase 1; mTOR: mammalian Target of Rapamycin; 14.3.3, “14” Fraction-“3.3” position of combined chromatography original protein purification; RAF: Rapidly Accelerated Fibrosarcoma proto-oncogene; B-RAF: RAF homolog B. For all other acronyms see text.
